# Income-related health inequality among rural residents in western China

**DOI:** 10.3389/fpubh.2022.1065808

**Published:** 2022-12-15

**Authors:** Chaofan Li, Chengxiang Tang

**Affiliations:** ^1^Centre for Health Management and Policy Research, School of Public Health, Cheeloo College of Medicine, Shandong University, Jinan, China; ^2^NHC Key Lab of Health Economics and Policy Research, Shandong University, Jinan, China; ^3^Centre for the Health Economy, Macquarie University, Sydney, NSW, Australia

**Keywords:** health inequality, income-related, China, western rural area, concentration index

## Abstract

**Objective:**

Health equality has drawn much public attention in both developed and developing countries. China, the largest developing country, has implemented a new round of health system reform to improve health equality since 2009. This study aims to examine the magnitude and sources of income-related health inequality in western rural regions of China.

**Methods:**

Data were obtained from the Survey of Rural Economic and Social Development in Western China conducted in 2014, in which 14,555 individuals from 5,299 households in 12 provinces were included. Health outcome variables of interest were self-rated health status, prevalence of chronic disease and four-week illness. Concentration index was calculated to assess magnitude of income-related health inequality, and nonlinear decomposition analysis was performed to identify the sources of health inequality.

**Results:**

The Concentration indexes for poor self-rated health status, prevalence of chronic disease and four-week illness were −0.0898 (*P*<0.001),−0.0860 (*P*<0.001) and −0.1284 (*P*<0.001), respectively. Income and education were two main sources of health inequality, accounting for about 25−50% and 15% contribution to the inequality. Ethnicity made <10% contribution to income-related health inequality, and enrollment in New Rural Cooperative Medical Scheme contributed to <1%.

**Conclusion:**

This study found slight income-related health inequality among rural residents in western China, implying that although China has made substantial progress in economic development and poverty alleviation, health inequality in western rural region should still be concerned by the government. To achieve health equality further, the Chinese government should not only strengthen its reimbursement mechanism of the current health insurance scheme to improve affordability of primary healthcare for residents in western rural regions, but also implement health poverty alleviation policies targeting socioeconomically vulnerable population and ethnic minorities in future.

## 1. Introduction

Achieving health equality is a widely recognized goal of public policy and health systems around the world (1). The third of Sustainable Development Goals put forward by the United Nations in 2015 calls for all countries to take action to ensure good health and wellbeing for all (2). During the past several decades, although the general population health status has improved, socioeconomic-related health inequality has remained persistent or even widened in both developed and developing countries (3–8). To achieve equality in health, the WHO proposed several initiatives at both the global and national levels, including identifying disadvantaged subgroups and focusing on low- and middle-income countries or regions (9, 10). According to the WHO, health inequality is defined as observable differences in health across subgroups (demographic, economic, ethnic, regional, social, etc.) within the overall population (11). In recent years, the WHO conducted a holistic review of social determinants of health to explore causes of health inequality and monitor its changing trends, and has suggested that income, education, and ethnicity were common social determinants of health (9).

China, as the largest middle-income country in the world, has achieved unprecedented progress in economic development since Reform and Opening-up in 1978. Although population health has gained continuing increasement thereafter, health inequality remained a serious issue, which posed challenges to the social and economic sustainable development (12, 13). In 2009, China started a new round of health system reform to improve population health and equality. Later, in 2016, the Chinese central government approved the <Healthy China 2030 Plan>, which provided a guideline for promoting health for all (14). Achieving health equality is a priority in this ambitious plan.

Researchers home and abroad have conducted many studies to examine the extent and causes of health inequality in China. They found that income-related self-reported health inequality remained or had increased during the past years (15–19), and inequality persisted in quality of life, prevalence of hypertension, maternal mortality, and child malnutrition (20–23). In contrast, only one study found decreased income-related inequality in self-rated health during the period of 2010 to 2014 (24). Pro-poor inequality was also remained during the period of the COVID-19 pandemic (25). The current literature consistently suggested that equality-oriented programs should be implemented to support vulnerable groups. However, conclusions regarding health inequality in China were only drawn based on studies among the overall population, which failed to show variations in subpopulations. As far as we know, there has been sparse research on income-related health inequality among residents in the western rural areas of China. Health inequality in this population worth further exploration for several reasons. First, rural western China is much less economically developed comparing to the rest areas. Second, western China is populated by ethnic minority groups. According to the 7th China National Census conducted in 2020, 70.2% of ethnic minority populations were concentrated in western China (26). Third, people living in the western rural regions have lower access to health resources and healthcare services than those residing in the eastern and central regions.

To fill the research gap, this study assessed the magnitude of income-related inequality in health status among adults and examined the socioeconomic determinants of health inequality, using data from a wide range of rural western regions and large-scale sampled residents. This study contributes to the understanding of health inequality in western rural regions and provides policy implications for improving health status and equality among vulnerable populations.

## 2. Materials and methods

### 2.1. Data and sample

Data were obtained from the Chinese Western Ethnicity Economic Survey (CWEES), which was conducted by the School of Economics, Southwestern Minzu University in 2014 (27). More details of this survey can be found in the book published by Zheng (28). The CWEES contains a wide range of information covering demographic characteristics, health status and social security, rural–urban migration, wellbeing, household income and expenditure, etc. Multiple stage sample methods were used to retrieve respondents from 12 provinces in the rural regions of western China. Two counties from each province were firstly selected using the purposive sampling method. Then, villages and households were drawn from the residents' registration system, using the probability proportionate to size sampling (PPS) method. All members of the sampled households were interviewed face-to-face by trained interviewers. Finally, a total of 23,172 individuals from 5,967 households were sampled and interviewed. For the present study, the inclusion criteria were: (1) aged 18 and older; (2) had no missing values in both health variables and independent variables. In total, 4,766 observations aged lower than 18 were excluded and 3,851 was excluded because of missing values. At last, 14,555 adults from 5,299 households were included for analysis.

### 2.2. Measurement

#### 2.2.1. Measurement of health status

The primary outcome variable of interest was self-assessed health. Self-assessed health is a powerful predictor of mortality and objective health status in the general population (29, 30), and has been widely used to measure socioeconomic inequalities in health (31–33). In this survey, self-assessed health was measured based on the question “How do you assess your health status?” with a five-point scale response: (1) very good; (2) good; (3) fair; (4) poor; (5) very poor. Following previous studies (24, 34), self-assessed health was dichotomized into two categories: (0) good (very good and good); (1) fair/poor (fair, poor, and very poor).

The second outcome variable was the prevalence of chronic disease, which was measured by the question “Are you currently suffering from chronic disease?” Answers to this question could be (0) no and (1) yes. Chronic diseases not only cause a mass of disability and premature deaths worldwide, but also lead to high financial burden (35, 36). Chronic diseases pose a heavy global public challenge, especially to developing countries and rural areas (37).

The third outcome variable was the prevalence of illness during a four-week period, which was measured by the question “Have you been ill during the last 4 weeks?” Answers to this question were: (0) no and (1) yes.

#### 2.2.2. Independent variables

Independent variables were factors that are widely known to be related to individuals' health status and thus to be associated with health inequality (38). Three domains of factors were included: demographic characteristics, socioeconomic status, and other variables. Demographic variables were gender and age. Age was categorized into six groups: (1) 18–24; (2) 25–34; (3) 35–44; (4) 45–54; (5) 55–64; (6) 65 years and above.

Socioeconomic variables included income, education level, and occupation status. Per capita household income was used to measure respondents' economic status and calculate the concentration index. Following a previous study (39), income was transformed into its natural logarithm value in multivariate regression models to decrease the variability of data. Education level was coded as five groups: (1) illiteracy; (2) primary school; (3) middle school; (4) senior school; and (5) undergraduate. Occupation status was divided into six groups: (1) agricultural work; (2) employed; (3) self-employed; (4) student; (5) retired; and (6) unemployed.

Other independent variables included ethnicity, health insurance enrolment, and marital status. Ethnicity was a dichotomous variable, (0) Han and (1) ethnic minorities. The New Rural Cooperative Medical Schemes (NRCMS) was a health insurance scheme launched for rural China in 2003, which covered about 97% of the rural population by 2013 (40). Enrolment in the NRCMS was coded as 1, and as 0 otherwise. Marital status was classified into four categories: (1) married; (2) separated/divorced; (3) widowed; and (4) unmarried.

### 2.3. Statistical analysis

Descriptive statistics was conducted to show basic characteristics of the respondents. The chi-square test was used to examine differences in health status across subgroups. The probit multivariate regression model was employed to examine the association between health outcomes and independent variables. Concentration curve and concentration index, widely used approaches based on the relative invariant principle, were used to measure health inequality. Following Wagstaff's guidelines, we first plotted a concentration curve to examine income-related inequality in health status (41). The concentration curve displayed the cumulative share of fair/poor self-assessed health (or presence of chronic disease and four-week illness) against the cumulative share of population, ranked by income from the lowest to the highest. The concentration curve intuitively displayed the distribution of health in the overall population. Second, we calculated the concentration index to quantify the degree of inequality in health, which was defined as twice the area between the concentration curve and the line of equality (the 45-degree diagonal line). Its value ranges from −1 to 1. When the concentration curve lies above the line of equality, it takes negative value and indicates that fair/poor self-assessed health is unevenly distributed among the poor, and vice versa. When the concentration curve coincides with the line of equality and the concentration index equals to zero, it means that there is no inequality in health. The covariance approach was used to compute the concentration index using “conindex” command in Stata (42):


(1) $$\begin{array}{l} C=\frac{2}{\mu }cov\left(h,\;r\right) \end{array}$$

where, *C* is the concentration index, *h* denotes the health variable and μ is its mean, *r* is the fractional rank of income, and *cov* means covariance between health variable and rank in income distribution. Robust standard errors clustering on household level was used to correct potential cluster sampling.

A decomposition method was employed to explain inequality in health and distinguish the contribution of various independent variables to the concentration index for the health variable (43). The concentration index for health variable could be written as:


(2) $$\begin{array}{l} C=\underset{i}{\sum }\frac{\beta_{i}^{m}\bar{x_{i}}}{\mu }C_{i}+\frac{GC_{\varepsilon }}{\mu } \end{array}$$

where, $\bar{x_{i}}$ is the mean of *i*_*th*_ independent variable, $\beta_{i}^{m}$ is its marginal effect and *C*_*i*_ is its concentration index, $\frac{\beta_{i}^{m}\bar{x_{i}}}{\mu }C_{i}$ means the contribution of *i*_*th*_ variable to the concentration index for health and $\frac{GC_{\varepsilon }}{\mu }$ is the contribution of the residual term. We examined income-related health inequality both among the total sample and separately among the Han and the ethnic minority subgroups. Furthermore, we used the relative index of inequality (RII) and slope index of inequality (SII) (44) to examine education-related health inequality. RII measured the ratios in prevalence of poor health, chronic disease, and four-week illness between the lowest educated and highest educated persons, while SII capture the absolute differences in prevalence (45).

All statistical analyses were conducted using Stata 15.1.

## 3. Results

### 3.1. Basic characteristics of respondents

Table 1 shows the basic characteristics of the 14,555 respondents. The majority of respondents (58.11%) assessed their health as good or very good, while about 41.89% rated their health as fair, poor, or very poor. Most of the adults reported having no chronic disease (73.04%) or illness during the last 4 weeks (82.90%). The proportion of respondents rating fair/poor health and the presence of chronic diseases or illness differed across subgroups. Specifically, respondents who were males, aged 18–24 years, Han ethnicity, and those who did not enroll in NRCMS were more likely to rate very good or good health comparing to their counterparts. Furthermore, the prevalence of chronic diseases and the prevalence of four-week illness in males, those aged 18–24 years, and respondents of Han ethnicity were lower than in other groups.

**Table 1:** Descriptive statistic for health status, demographic, socioeconomic and other characteristics of the Chinese western rural adults in 2014 (*N* = 14,555).

**Variables**	**Total, *N* (%)**	**Self-rated health status** ^**#**^		**Chronic disease** ^**#**^		**Four-week Illness** ^**#**^	
		**Good, *N* (%)**	**Fair or poor, *N* (%)**	**No, *N* (%)**	**Yes, *N* (%)**	**No, *N* (%)**	**Yes, *N* (%)**
Total	1,4555	8,458 (58.11)	6,097 (41.89)	1,0631 (73.04)	3,924 (26.96)	1,2066 (82.90)	2,489 (17.10)
**Gender**							
Female	6,972 (47.90)	3,767 (54.03)	3,205 (45.97)	4,870 (69.85)	2,102 (30.15)	5,581 (80.05)	1,391 (19.95)
Male	7,583 (52.10)	4,691 (61.86)	2,892 (38.14)	5,761 (75.97)	1,822 (24.03)	6,485 (85.52)	1,098 (14.48)
**Age groups**							
18−24	1,918 (13.18)	1,604 (83.63)	314 (16.37)	1,837 (95.78)	81 (4.22)	1,824 (95.10)	94 (4.90)
25−34	2,793 (19.19)	2,133 (76.37)	660 (23.63)	2,529 (90.55)	264 (9.45)	2,572 (92.09)	221 (7.91)
35−44	3,062 (21.04)	1,867 (60.97)	1,195 (39.03)	2,425 (79.20)	637 (20.80)	2,637 (86.12)	425 (13.88)
45−54	2,811 (19.31)	1,489 (52.97)	1,322 (47.03)	1,942 (69.09)	869 (30.91)	2,285 (81.29)	526 (18.71)
55−64	2,151 (14.78)	821 (38.17)	1,330 (61.83)	1,132 (52.63)	1,019 (47.37)	1,546 (71.87)	605 (28.13)
65+	1,820 (12.50)	544 (29.89)	1,276 (70.11)	766 (42.09)	1,054 (57.91)	1,202 (66.04)	618 (33.96)
**Marital status**							
Married	1,1426 (78.50)	6,338 (55.47)	5,088 (44.53)	8,057 (70.51)	3,369 (29.49)	9,348 (81.81)	2,078 (18.19)
Separated/divorced	154 (1.06)	84 (54.55)	70 (45.45)	124 (80.52)	30 (19.48)	129 (83.77)	25 (16.23)
Widowed	737 (5.06)	256 (34.74)	481 (65.26)	384 (52.10)	353 (47.90)	500 (67.84)	237 (32.16)
Unmarried	2,238 (15.38)	1,780 (79.54)	458 (20.46)	2,066 (92.31)	172 (7.69)	2,089 (93.34)	149 (6.66)
**Education**							
Illiteracy	3,155 (21.68)	1,232 (39.05)	1,923 (60.95)	1,723 (54.61)	1,432 (45.39)	2,245 (71.16)	910 (28.84)
Primary school	4,490 (30.85)	2,315 (51.56)	2,175 (48.44)	3,060 (68.15)	1,430 (31.85)	3,589 (79.93)	901 (20.07)
Middle school	4,497 (30.90)	3,018 (67.11)	1,479 (32.89)	3,675 (81.72)	822 (18.28)	4,001 (88.97)	496 (11.03)
Senior school	1,566 (10.76)	1,175 (75.03)	391 (24.97)	1,367 (87.29)	199 (12.71)	1,428 (91.19)	138 (8.81)
Undergraduate	847 (5.82)	718 (84.77)	129 (15.23)	806 (95.16)	41 (4.84)	803 (94.81)	44 (5.19)
**Employment status**							
Agricultural work	9,827 (67.52)	5,564 (56.62)	4,263 (43.38)	7,152 (72.78)	2,675 (27.22)	8,100 (82.43)	1,727 (17.57)
Employed	1,322 (9.08)	1,022 (77.31)	300 (22.69)	1,177 (89.03)	145 (10.97)	1,207 (91.30)	115 (8.70)
Self-employed	684 (4.70)	469 (68.57)	215 (31.43)	569 (83.19)	115 (16.81)	616 (90.06)	68 (9.94)
Student	658 (4.52)	541 (82.22)	117 (17.78)	633 (96.20)	25 (3.80)	622 (94.53)	36 (5.47)
Retired	282 (1.94)	124 (43.97)	158 (56.03)	145 (51.42)	137 (48.58)	204 (72.34)	78 (27.66)
Unemployed	1,782 (12.24)	738 (41.41)	1,044 (58.59)	955 (53.59)	827 (46.41)	1,317 (73.91)	465 (26.09)
**Ethnicity**							
Han	6,335 (43.52)	3,933 (62.08)	2,402 (37.92)	4,641 (73.26)	1,694 (26.74)	5,517 (87.09)	818 (12.91)
Minority	8,220 (56.48)	4,525 (55.05)	3,695 (44.95)	5,990 (72.87)	2,230 (27.13)	6,549 (79.67)	1,671 (20.33)
**NRCMS**							
No	671 (4.61)	430 (64.08)	241 (35.92)	533 (79.43)	138 (20.57)	575 (85.69)	96 (14.31)
Yes	13,884 (95.39)	8,028 (57.82)	5,856 (42.18)	10,098 (72.73)	3,786 (27.27)	11,491 (82.76)	2,393 (17.24)

NRCMS, New Rural Cooperative Medical Scheme.

# Chi-square test was used to compare differences among subgroups. All tests were significant at the p = 0.01 level, except for the difference in prevalence of four-week illness between the enrollees of NRCMS and the non-enrollees.

About 50% of the respondents had an education level of lower than primary school, and the cumulative percentage of senior school and undergraduate was ~15%. Respondents with lower education levels were more likely to rate their health as fair/poor than those who had higher education levels. Similarly, the prevalence of chronic disease and four-week illness was higher in the low-educated groups than that in high-educated groups. As shown in Supplementary Table 1, the SIIs for poor health status, chronic disease, and four-week illness between the lowest educated and the highest educated were significantly negative, and the RIIs were significantly positive at 0.001 level. About 68% of the respondents were engaged in agricultural work, while only 18% were employed, self-employed or were students. Fourteen percent of the respondents were retired or unemployed, these respondents were more likely to rate their health status as fair/poor or report higher presence of chronic diseases and four-week illness than other groups did.

### 3.2. Concentration index for health status

**Figure 1:**
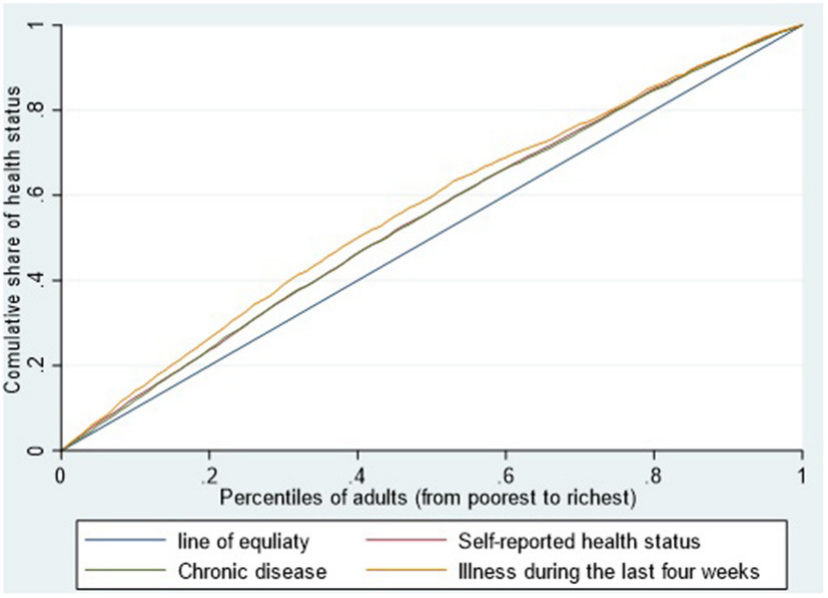
The concentration curve of health status among Chinese western rural adults in 2014 (N = 14,555).

Table 2 displays the concentration indexes for the three outcome variables. The concentration indexes for self-rated health status, prevalence of chronic disease, and morbidity of illness were −0.0898, −0.0860, and −0.1284, respectively, all were significant at 0.001 level. Figure 1 intuitively displays the distribution of health in relation to income. The concentration curves for the three health variables lie above the equality lines. The significantly negative CCI values and the location of concentration curves indicate that fair/poor self-rated health, chronic disease, and four-week illness were unevenly concentrated among the poor. In other words, the poor were more likely to have worth health than the rich. Similarly, the concentration indexes for the three health variables among the Han or among the minorities were all significantly negative at 0.001 level.

**Table 2:** Concentration index for health status of Chinese western rural adults in 2014 (*N* = 14,555).

	**Self-assessed health status**		**Chronic disease**		**Four-week illness**	
	**CCI (Robust SE)**	***P***	**CCI (Robust SE)**	***P***	**CCI (Robust SE)**	***P***
Total population	−0.0898 (0.0075)	<0.001	−0.0860 (0.0092)	<0.001	−0.1284 (0.0128)	<0.001
Han	−0.1089 (0.0120)	<0.001	−0.1042 (0.0137)	<0.001	−0.1105 (0.0227)	<0.001
Minority	−0.0698 (0.0093)	<0.001	−0.0718 (0.0121)	<0.001	−0.1176 (0.0149)	<0.001

CCI, concentration index; SE, standard error.

### 3.3. Decomposition of concentration index for health status

Table 3 displays the contribution of each factor to inequality in health. Demographic, socioeconomic, and other variables explained the majority of the inequality, ~99% of self-assessed health, 94% of chronic disease, and 90% of four-week illness. The significantly negative coefficients of income and education level indicate that respondents with higher income or better education were less likely to rate their health status as fair/poor or report chronic disease than respondents with lower income or lower education. According to the decomposition results, socioeconomic status was the main source of inequality. Income made 52, 25, and 40% of the contribution to inequality in self-assessed health, chronic disease, and four-week illness, respectively. Education also made about 15–23% contribution to pro-poor inequality, except for primary school, which only made about 1–3% negative contribution. Employment status made lower than 10% contribution to inequality in health; however, not all employment statuses were significant. As for other factors, ethnicity was significantly associated with health outcomes. The corresponding coefficients of ethnicity for self-rated health status, prevalence of chronic disease, and four-week illness were 0.21 (*P* < 0.001), 0.05 (*P* < 0.05) and 0.33 (*P* < 0.001). Comparing to the Han, people of minority ethnicities were more likely to rate a fair/poor health status and report four-week illness. Ethnicity made about 7, 2, and 11% contribution to inequality in self-rated health status, prevalence of chronic disease, and four-week illness, respectively. With respect to health insurance scheme, NRCMS was significantly positively associated with the presence of chronic disease (*β* = 0.14, *P* < 0.05) but not related to self-assessed health status and four-week illness. The contribution of NRCMS to health inequality was <1%, ranging from 0.18 to 0.56%.

**Table 3:** Decomposition of concentration index for health status of Chinese western rural adults in 2014 (*N* = 14,555).

**Variables**	**CCI_k_**	**Self–rated health status**		**Chronic disease**		**Four–week illness**	
		**Coefficient**	**Contribution (%)**	**Coefficient**	**Contribution (%)**	**Coefficient**	**Contribution (%)**
**Gender (ref. Female)**							
Male	0.01	−0.16^***^	0.63	−0.13^***^	0.64	−0.17^***^	0.69
**Age (ref. 18–24)**							
25–34	0.04	0.23^***^	−1.64	0.31^***^	−2.91	0.22^p**^	−1.63
35–44	0.01	0.61^***^	−1.46	0.74^***^	−2.46	0.50^***^	−1.37
45–54	0.05	0.83^***^	−8.62	1.05^***^	−15.98	0.71^***^	−9.10
55–64	−0.06	1.14^***^	9.33	1.40^***^	17.82	0.97^***^	11.02
65+	−0.13	1.31^***^	20.02	1.59^***^	38.92	1.09^***^	24.70
**Marital (ref. Married)**							
Separated/divorced	0.08	0.13	−0.11	−0.22	0.21	0.03	−0.03
Widowed	−0.06	−0.01	−0.03	−0.16^p**^	−0.59	0.02	0.06
Unmarried	0.04	−0.01	0.07	−0.06	0.44	−0.03	0.16
**Education (ref. Illiteracy)**							
Primary school	−0.05	−0.09^p**^	−1.49	−0.13^***^	−2.65	−0.08^p*^	−1.25
Middle school	0.07	−0.24^***^	5.46	−0.30^***^	8.40	−0.24^***^	5.38
Senior school	0.15	−0.38^***^	5.86	−0.43^***^	7.60	−0.29^***^	4.13
Undergraduate	0.23	−0.49^***^	6.09	−0.60^***^	8.05	−0.38^***^	4.23
**Occupation (ref. Agricultural work)**							
Employed	0.26	−0.12^p**^	2.78	−0.13^p*^	3.83	−0.001	0.03
Self–employed	0.25	−0.10	1.16	−0.14^p*^	1.98	−0.14^p*^	1.59
Student	0.03	0.17^p*^	−0.20	0.02	−0.02	0.14	−0.17
Retired	0.19	−0.10	0.40	0.09	−0.45	0.04	−0.16
Unemployed	−0.08	0.17^***^	1.79	0.29^***^	4.22	0.08^p*^	0.92
**Ethnicity (ref. Han)**							
Minority	−0.06	0.21^***^	7.03	0.05^p*^	2.06	0.33^***^	10.67
**NRCMS (ref. No)**							
Yes	−0.003	0.05	0.18	0.14^p*^	0.56	0.06	0.18
**Ln per capita income**	0.07	−0.18^***^	51.53	−0.07^p**^	24.83	−0.14^***^	39.52
Total explained		98.79		94.47		89.57	
Residual terms		1.21		5.53		10.43	

CCI_k_, concentration index for variable k; ref., reference to; NRCMS, New Rural Cooperative Medical Scheme.

* P < 0.05;

** P < 0.01;

*** P < 0.001.

## 4. Discussion

Using large scale of samples, this study examined the magnitude and source of health inequality among adults in rural western China based on the concentration index approach. We found that all concentration indexes for the three health status variables were negative but small, indicating that slight pro-poor income-related inequality existed in health. In addition, nonlinear decomposition of CCI demonstrated that income and education level were the main sources of health inequality. Besides, ethnicity and NRCMS made smaller contribution to health inequality.

### 4.1. Magnitude of income-related health inequality

Comparing to previous findings, our study found that pro-poor income-related health inequality in rural regions of western China was attenuated. The concentration index for self-assessed health status among our sample was lower than that for the total population (29). Moreover, we found that pro-poor inequality existed in the prevalence of self-reported chronic disease. This finding was in contrast to several previous studies, which found pro-rich inequality in prevalence of self-reported hypertension (22), diabetes (46), or other chronic disease (47, 48) among rural residents. However, Cao found that the prevalence of tested hypertension was unequally concentrated among the poor respondents in China (49), which as consistent with our findings. We also found pro-poor inequality in the prevalence of four-week illness. Similarly, small and negative concentration indexes for the prevalence of four-week illness and prevalence of chronic disease were found among rural residents in Yunnan province, an ethnic frontier region in southwest China (50). In sum, the negative CCI values for the three health variables in our study provide robust evidences for attenuated pro-poor health inequality among rural residents in western China.

### 4.2. Source of income-related health inequality

Firstly, according to the decomposition results, the main driver of pro-poor income-related health inequality was income, followed by education. These findings are in accordance with findings from developed countries (39, 51). Several reasons may explain why income and education contribute much to health inequality. First, socioeconomic disparity may directly result in differences in health determinants, such as nutrition, diet, and eating habit (52). People with higher income could have better access to healthy food and nutrition than those making less income. Second, socioeconomic status is related to physical activities and health behaviors, such as heavy physical work, smoking, and drinking (50). Unhealthy behaviors may lead to high prevalence of chronic disease and four-week illness. Third, the rich usually have better access to health care and health literacy to seek healthcare when they are ill than the poor (25). Such inequity in healthcare could also exacerbate health inequality. The finding that health inequality is mainly due to disparities in socioeconomic determinants of health highlights that the government should not just give priority to economic development, but also pay attention to equality in income distribution and resource allocation, especially focusing on poverty alleviation policies targeting vulnerable groups.

Secondly, it is important to note that ethnicity makes about 2–11% contribution to pro-poor income-related health inequality among our sample. The ethnic minorities not only had lower income than the Han population, but also reported poorer health status. Such inequality was supported by study conducted by Castro Campos et al. (53), Ouyang and Pinstrup-Andersen (54), and Wang et al. (55). According to our descriptive and regression results, there was a small difference in the prevalence of chronic disease between ethnic minorities and the Han population; however, gaps existed in self-reported health and the prevalence of four-week illness. Residential environment of ethnic minorities is an important risk factor of poor self-reported health and four-week illness (56). Ethnic minorities usually live in remote and mountainous regions with a harsh natural environment, which may contribute to their poor health status and high prevalence of illness (50). Furthermore, remote rural residential environments are always accompanied by poorer health infrastructure and fewer health professionals (57). Thus, the ethnic minorities usually have many barriers in utilizing healthcare, such as geographical accessibility, cultural acceptability, financial affordability, and health resource availability (58). Once they are ill, they face more difficulties in seeking high-quality medical treatment services than people living in the eastern and central regions of China.

Thirdly, we found that NRCMS also made a low percentage of contribution to pro-poor income-related health inequality. This finding was consistent with previous study which demonstrated that China's health insurance scheme could lead to health inequality (17). A study in Canada and America also supported that health insurance enrolment contributed to income-related health inequality (38). In this study, we found that the NRCMS contributed to inequality in the prevalence of chronic disease but not in self-rated health or four-week illness. One possible explanation could be that the benefit package of NRCMS did not cover treatment for chronic diseases. As previous studies found, the NRCMS reimbursement policy had little effect on healthcare utilization and financial protection for respondents with chronic disease (59, 60). This finding implies that the NRCMS should be strengthen with respect to reimbursement policy toward chronic disease preventive and treatment care.

### 4.3. Policy implications

This study contributes to the understanding of the situation and sources of health inequality in rural regions of western China. These findings have several important policy implications. Firstly, the high contribution of income to health inequality illustrates that income is still the main contributor to inequality. To narrow the disparity in health status across income, the government should continue to implement targeted poverty alleviation policies, which have been proofed an effective strategy to attenuate health inequality (61). Based on the order of contributions and each factor made, people with low income, ethnic minorities, people with low education attainment, and those with chronic diseases should be primarily targeted by poverty alleviation policies. Secondly, to improve health of the minorities, more health promotion measures should be taken, such as to improve access to high-quality primary healthcare, acceptability, and health literacy. Furthermore, healthcare providers may provide telehealth care for the western rural residents, especially for those living in mountainous regions with geographic barriers (61, 62). Thirdly, to improve the contribution of NRCMS to promote health equality, the government should improve the affordability of healthcare by strengthening the health insurance reimbursement policies on both preventive and outpatient care for patients with chronic diseases.

### 4.4. Strengths and limitations

This research has two notable strengths. First, the data were obtained from a large-scale survey conducted in rural regions of western China. To the best of our knowledge, it is the first survey focusing on rural western China and covers a wide range of regions (12 provinces and 24 counites) and residents. Based on the large-scale samples, we could validly examine income-related health inequality among residents living in rural regions of western China. Second, three subjective health outcomes were used to measure health status, which could provide robust evidence of inequality in health.

However, our study has four limitations. First, the cross-sectional survey was conducted in 2014. It cannot reflect the changing trends of health inequality in recent years. Further research on health inequality could use longitudinal data, if available, to examine the changing trends of inequality in the fight against poverty progress in China during the past 8 years. Second, the decomposition of CCI with cross-sectional survey data could display the contribution of socioeconomic characteristics and other factors to health inequality; however, it could not verify the causal relationship between independent variables and health status. Causal inference methods, such as difference-in-difference, are needed to examine the causal link between public health policy and health (63). Third, the measurement of health status was self-rated or reported. The rich may have higher health expectations and hence report poorer health and higher prevalence of chronic disease or illness, which may lead to underestimation of the magnitude of health inequality in reality (34). Future research may use objective indicators of health, such as biomarkers, mortality, nutrition and so on. Fourth, 3,851 observations, which accounted for 21% of the sampled adults, were excluded for missing values. The missing values were more concentrated in older adults, socioeconomic deprived, and those with poor health status. Thus, the missing data may lead to underestimated bias in health inequality.

## 5. Conclusions

This study found evidence that there remains an attenuated income-related health inequality among adults in Chinese western rural areas. To eliminate health inequality and achieve health for all, a targeted poverty alleviation policy and equal education opportunity program targeting vulnerable groups should be implemented continuously in the future. Moreover, a targeted policy toward ethnic minorities living in remote areas should be designed and implemented to promote equal access to high-quality primary healthcare. Furthermore, the government should also strengthen the health insurance reimbursement policies for residents with chronic disease to promote health equality.

## Data availability statement

The original contributions presented in the study are included in the article/Supplementary material, further inquiries can be directed to the corresponding author.

## Author contributions

CL and CT participated in concept, study design, data analysis, interpretation, and writing the manuscript. All authors reviewed and approved the final manuscript.
