# Supplementary material for: Income-related health inequality among rural residents in western China
Source: Front Public Health. 2022 Dec 15;10:1065808. doi: 10.3389/fpubh.2022.1065808 (PMC9797679; doi:10.3389/fpubh.2022.1065808)
Supplement: Supplementary file 1 [file Data_Sheet_1.PDF]

Supplementary Table 1. Education-related health inequality among Chinese western rural adults in 2014 (N=14,555)

	Self-rated health status		Chronic disease		Four-week illness	
	Index value (Robust SE)	*P*	Index value (Robust SE)	*P*	Index value (Robust SE)	*P*
Slope Index of Inequality	-0.4669 (0.0138)	<0.001	-0.4290 (0.0125)	<0.001	-0.2719 (0.0121)	<0.001
Relative Index of Inequality	0.2997 (0.0131)	<0.001	0.1838 (0.0101)	<0.001	0.2039 (0.0146)	<0.001

SE, standard error.
